# Protracted Bacterial Bronchitis in Children: Clinical Presentation, Diagnostic Evaluation, and Evidence-Based Management

**DOI:** 10.7759/cureus.117943

**Published:** 2026-10-14

**Authors:** Mishal Al-Rsheedi, Khalid Al-Shamrani, Faisal Al-Shamrani, Abdullah Al-Shamrani

**Affiliations:** 1 Pediatrics, Prince Sultan Military Medical City, Riyadh, SAU; 2 Medical School, Almaarefa University, Riyadh, SAU; 3 Medical School, Alfaisal University, Riyadh, SAU; 4 Pediatrics, Alfaisal University, Riyadh, SAU

**Keywords:** amoxicillin-clavulanate, bronchiectasis, bronchoscopy, children, chronic wet cough, protracted bacterial bronchitis

## Abstract

Protracted bacterial bronchitis (PBB) is a treatable cause of chronic wet cough in children but is frequently missed. Unrecognized children often cycle through repeat asthma treatment, short antibiotic courses that do not address the infection, or open-ended reassurance without further evaluation. This review summarizes the clinical presentation, diagnostic criteria, differential diagnosis, treatment, and follow-up of PBB in children. We conducted a targeted narrative review (PubMed, through September 2026) of clinical trials, longitudinal studies, systematic reviews, and professional-society guidance, distinguishing evidence for antibiotic treatment from limited observational evidence linking recurrent PBB to bronchiectasis. Clinicians should suspect PBB in a child with a daily wet cough lasting more than four weeks and no findings indicating another cause. Resolution after an appropriate antibiotic course supports the diagnosis. Amoxicillin-clavulanate remains the usual first-line treatment, typically for two weeks initially, with reassessment and selective extension to four weeks if the child has not cleared. Further investigation is warranted when symptoms persist despite adequate treatment, recurrence is frequent, or specific warning signs appear. In an otherwise typical, treatment-responsive case, bronchoscopy and chest computed tomography (CT) are not routine first steps. Management hinges on recognizing the wet-cough phenotype, giving an adequate therapeutic trial, confirming resolution, and investigating promptly when treatment fails. Antibiotics appear to improve cough resolution, but this rests on a small number of trials with limited certainty, and evidence that treatment itself prevents bronchiectasis remains thin.

## Introduction and background

A child with a daily wet cough lasting more than four weeks should not be dismissed as just another childhood cough. Protracted bacterial bronchitis (PBB) is among the commonest causes of chronic wet cough in children [1,2]. In one Western Australian tertiary service, it accounted for 37.9% of children referred with chronic cough, yet asthma treatment had been prescribed for 34.0% of children with PBB, and only 13.5% of those still coughing at first review had received four weeks of antibiotics [3]. Early recognition and effective treatment of chronic wet cough are emphasized in current guidance [4]. PBB matters most in young children whose cough lingers after a routine respiratory infection, even when they seem well. The clinical challenge is distinguishing an infection that will respond to antibiotics from asthma, recurrent viral illness, or an airway-clearance problem. PBB is not simply another label for childhood bronchitis, and it is not the same as established bronchiectasis. Its definition combines the clinical pattern with the child's response to treatment, so suspected PBB is often the right starting label and becomes a supported diagnosis only once the wet cough clears. For clarity, chronic cough denotes daily cough lasting more than four weeks; chronic suppurative lung disease (CSLD) describes chronic wet cough with features of persistent airway infection that may precede bronchiectasis, which is irreversible airway dilatation confirmed on chest CT.

This narrative review summarizes the clinical presentation, diagnostic approach, differential diagnosis, treatment, and follow-up of PBB in children, with attention to practical management and dosing, and complements earlier overviews [2,5,6] by integrating the most recent trial and cohort evidence on antibiotic duration, lower-airway sampling, and long-term outcomes.

## Review

Methods

This was a targeted narrative review. PubMed was searched without a start-date restriction through September 2026, and the reference lists of key publications, clinical guidelines, and relevant studies were examined. Search terms, combined with Boolean operators, included PBB, chronic wet cough, children, antibiotics, bronchoscopy, and bronchiectasis. One author screened titles and abstracts and read the full text of potentially relevant English-language publications on children; the selection was reviewed by a second author, and disagreements were resolved by discussion. Priority was given to randomized trials, systematic reviews, and professional-society guidance, supplemented by cohort and cross-sectional studies where trial evidence was lacking, as for microbiology, prognosis, and bronchiectasis risk. Earlier foundational studies were retained when they established definitions or natural history, and newer studies were preferred for treatment duration, airway sampling, and long-term outcomes. Embase and the Cochrane Library were not systematically searched (see Limitations). This was not a systematic review; therefore, no formal risk-of-bias or quality assessment was performed, and the selection of evidence was based on clinical relevance and author judgment.

Definitions

The definitions most often used in clinical practice and research are summarized in Table 1.

**Table 1:** Commonly used definitions PBB: protracted bacterial bronchitis Definitions are based on the European Respiratory Society statement [2], the CHEST guideline [6], and the Royal Children's Hospital Melbourne and Perth Children's Hospital guidelines [7,8].

Term	Definition
Clinical PBB	Daily wet cough lasting >4 weeks; no specific findings indicating another cause; cough resolves following an appropriate antibiotic course, usually within two weeks.
Microbiologically confirmed PBB	The compatible clinical syndrome with a significant lower-airway bacterial culture, usually from bronchoalveolar lavage, conventionally ≥10⁴ colony-forming units/mL, and an appropriate treatment response.
PBB-extended	The cough requires approximately four weeks of appropriate antibiotic treatment to resolve.
Recurrent PBB	More than three PBB episodes within 12 months.

These definitions follow the European Respiratory Society (ERS) statement [2] and the American College of Chest Physicians (CHEST) guideline [6], though some broader definitions allow resolution within two to four weeks rather than limiting clinical PBB to a two-week response. The threshold of more than three episodes in 12 months for recurrent PBB is also used by the Royal Children's Hospital and Perth Children's Hospital guidelines [7,8]. Recurrence must be carefully distinguished from persistence. If a child's cough never really goes away, calling each flare a "new episode" is misleading; that is persistent disease and should prompt a review of treatment adequacy and diagnosis. In a prospective cohort, almost half of children with PBB had recurrent episodes [9], which is why confirming resolution matters more than assuming spontaneous improvement.

Microbiology and pathophysiology

PBB involves bacterial infection with neutrophilic airway inflammation [10]. The organisms most often recovered are non-typeable *Haemophilus influenzae*, *Streptococcus pneumoniae*, and *Moraxella catarrhalis* [2,11]. Respiratory viruses can also be found, but their presence does not rule out a concurrent bacterial process in the lower airway. Basic immune workups are usually unremarkable in typical cases [9]. Airway malacia may coexist and impair secretion clearance, possibly contributing to persistence or recurrence [12]. However, the high rates of malacia observed in bronchoscopy series should not be extrapolated to all children with PBB, as children undergoing bronchoscopy are a selected group.

A 2022 cross-sectional study of archived bronchoalveolar lavage specimens detected lower-airway biofilms in 36% of children with PBB and 41% of children with bronchiectasis, with biofilms more frequent when lower-airway infection was present [13]. This suggests a mechanism by which bacteria may persist in the lower airway. It does not mean that every child with PBB has a biofilm, or that prolonged antibiotics or experimental antibiofilm treatments should become routine. Lavage microbiota profiling found that neutrophilic inflammation was associated with several distinct microbiota profiles and with bacterial biomass rather than with any single pathogenic species, so the contribution of the wider microbiota to recurrence and progression remains to be defined [14].

Clinical presentation

The Typical Presentation

The hallmark symptom is a wet-sounding cough every day, often throughout the day and sometimes at night. Young children tend to swallow respiratory secretions instead of coughing them up, so absence of visible sputum does not mean the cough is dry. Most affected children stay active, grow well, and do not have persistent fevers or respiratory distress. The examination may be normal or show nonspecific rattling sounds. A normal chest radiograph, or one with minor perihilar or peribronchial changes, is consistent with PBB. Beyond the infection, chronic wet cough takes a real toll on families: repeated medical consultations and parental stress that ease when the cough stops [15], and, in practice, disrupted sleep and missed school or daycare. Cough-specific quality of life is reduced, and societal costs are substantial [16]. A validated parent-proxy cough-specific quality-of-life questionnaire [17] can help clinicians gauge treatment response beyond counting symptoms.

Wheeze and Confusion With Asthma

Wheeze or noisy breathing can show up alongside PBB, and parents sometimes describe secretions as "wheezing." However, persistent wet cough on its own is not evidence of asthma. If repeated bronchodilator or inhaled corticosteroid treatment is not working, that is a cue to reconsider the diagnosis, not to escalate the same approach [11,18,19]. PBB and asthma are not mutually exclusive. A five-year follow-up study found clinician-diagnosed asthma in 27.1% of children who had had PBB [20]. That is overlap, not evidence that PBB causes asthma; variable airflow limitation and other asthma-compatible features still need their own assessment [1,20].

Features Inconsistent With Uncomplicated PBB

Hypoxemia, marked respiratory distress, hemoptysis, persistent fever, poor growth, digital clubbing, focal chest findings, or recurrent pneumonia all call for broader evaluation. Sudden choking with one-sided findings needs urgent assessment for foreign-body aspiration; that is not something to wait out with a PBB treatment trial [19,21].

Differential diagnosis

Table 2 lists the main alternative diagnoses and the clues that should prompt consideration of each.

**Table 2:** Important alternatives and diagnostic clues The table was compiled from the multicenter chronic-cough study [1], the CHEST chronic-cough guideline [19], the European Respiratory Society (ERS) chronic-cough guideline [21], and the ERS bronchiectasis guideline [22].

Diagnosis	Features that should raise suspicion
Repeated viral respiratory infections or post-infectious cough (including pertussis)	Distinct illnesses with genuine improvement or cough-free intervals; avoid assuming several uninterrupted weeks of wet cough are simply separate colds. Post-infectious cough follows a clear acute infection, improves steadily over time, and is not expected to respond to antibiotics; reassess if a daily wet cough persists beyond four weeks. Consider pertussis with paroxysmal cough, post-tussive vomiting, an inspiratory whoop, apneas in young infants, incomplete vaccination, or known exposure; antibiotics are unlikely to help unless started very early.
Asthma	Variable wheeze, breathlessness, exercise-related symptoms, and demonstrable bronchodilator or controller response. Wet cough can coexist but is not diagnostic.
Foreign-body aspiration	Abrupt onset, choking, asymmetric air entry, or localized wheeze. A normal radiograph does not reliably exclude it.
Aspiration/swallowing dysfunction	Coughing or choking with feeds, a wet voice after feeding, neurological impairment, or recurrent dependent-lung disease.
Airway malacia or structural obstruction	Early onset, brassy or persistent noisy breathing, positional symptoms, or difficulty clearing secretions.
Bronchiectasis/chronic suppurative lung disease	Persistent or recurrent wet cough despite adequate treatment, recurrent pneumonia, clubbing, or persistent radiographic abnormalities.
Cystic fibrosis or primary ciliary dyskinesia	Symptoms from early life, poor growth or malabsorption, persistent upper-airway disease, neonatal respiratory problems, or relevant family history.
Immunodeficiency or another chronic infection	Unusually severe or recurrent infections, infections outside the respiratory tract, systemic illness, or relevant tuberculosis exposure or HIV.

These are clues to watch for, not a checklist that must be fully satisfied before pursuing further investigation [1,19,21,22].

Diagnostic approach

Establish the Cough Pattern

Begin with a check on the cough pattern: has it really been wet every day, how long has it persisted, and have there been real cough-free periods? Ask about onset, feeding associations, previous pneumonia, growth, environmental exposures, past antibiotic courses, and the child's response to each. A caregiver recording can help when the child does not cough during consultation. It is important to verify whether earlier antibiotics were appropriate for the likely organisms and taken for the full prescribed duration. "The antibiotics did not work" tells little without knowing the agent, dose, duration, adherence, and degree of improvement.

Initial Investigations

A chest radiograph and, in children older than about six years, spirometry are standard in a pediatric chronic-cough workup, though normal results do not exclude PBB [4,19]. In a typical case without warning signs, extensive laboratory testing, bronchoscopy, and CT are usually unnecessary before an initial treatment trial. Track response with a simple daily record of wet cough, nighttime disturbances, and activity. The goal is resolution, not just "some improvement." Response to treatment is diagnostically useful but not fully specific; other bacterial airway disorders can also improve temporarily with antibiotics.

Microbiological Sampling

An expectorated sputum sample, when obtainable, helps guide treatment. A throat or nasal culture, however, cannot be assumed to reflect what is happening in the lower airway. Children with persistent or recurrent disease may need flexible bronchoscopy with bronchoalveolar lavage, both for culture and to assess airway anatomy [6,18,23,24].

The 2026 multicenter CLASSIC-PBB study compared cough swabs and induced sputum against bronchoscopic lavage in children referred for bronchoscopy [23]. Among 135 children who underwent lavage, cough swab and induced sputum results were discordant with six-lobe lavage in 74% and 71% of children, respectively, and the false-negative rate was 67% for cough swabs and 27% for induced sputum. Neither method was a dependable replacement when comprehensive lower-airway sampling was needed. Induced sputum can be informative in selected children, but a negative result should not reassure. This conclusion does not support bronchoscopy in every uncomplicated first episode.

When Should Bronchoscopy or CT Be Considered?

Further investigation makes sense when wet cough persists despite four weeks of appropriate antibiotics, when episodes are frequent, or when clinical warning signs appear. Bronchoscopy evaluates airway abnormalities, foreign material, secretions, and microbiology; pediatric-protocol chest CT assesses for suspected bronchiectasis. Which to choose, and in what order, depends on the specific clinical question [6,22]. One retrospective study of children already selected for CT because of chronic wet cough found antibiotic nonresponse strongly associated with bronchiectasis, though even a positive antibiotic response did not fully exclude it. The very high disease frequency seen in that selected group should not be mistaken for the risk facing an otherwise healthy child having a first PBB episode [25].

Evidence supporting antibiotic treatment

The principal randomized-trial and systematic-review evidence is summarized in Table 3.

**Table 3:** Principal treatment studies DACS: Duration of Amoxicillin-Clavulanate for Protracted Bacterial Bronchitis in Children trial; PBB: protracted bacterial bronchitis The studies summarized are those cited in the first column [26-28].

Study	Design and findings	Clinical interpretation
Marchant et al., 2012 [26]	Placebo-controlled trial of 50 children with wet cough lasting >3 weeks. Two weeks of amoxicillin-clavulanate achieved the study-defined cough-resolution outcome in 48% versus 16% with placebo.	Supports antibiotic efficacy. The trial used an older duration threshold and a composite response definition, not exclusively complete cough disappearance.
Cochrane review, 2018 [27]	Three trials involving 190 children. Antibiotics improved cough outcomes; the number needed to treat for one additional beneficial outcome was approximately 3.	Supports treatment of appropriately selected prolonged wet cough; long-term outcomes and adverse-event estimates remained limited.
DACS trial, 2021 [28]	Double-blind trial of 106 children (52 in the four-week group, 54 in the two-week group) comparing four weeks of amoxicillin-clavulanate with two weeks followed by two weeks of placebo. Day-28 clinical cure (primary outcome) occurred in 62% (32/52) with four weeks vs 70% (38/54) with two weeks (adjusted RR 0.87, 95% CI 0.60-1.28; p=0.49).	Four weeks was not superior to two weeks for the primary outcome; the difference was not significant.
DACS secondary outcome [28]	Median time to next wet-cough exacerbation was 150 days (IQR 38-181) with four weeks vs 36 days (IQR 15-181) with two weeks (adjusted HR 0.47, 95% CI 0.25-0.90; p=0.02). Recurrence of PBB at six months (53% vs 74%) was not significant (p=0.07).	Secondary, exploratory outcome. A longer course may benefit selected children, but this does not establish four weeks as necessary for every episode.

The principal data come from two randomized trials, including the Duration of Amoxicillin-Clavulanate for PBB trial (DACS), and a Cochrane review [26-28]. Together, these findings support starting with a two-week course with planned reassessment, rather than defaulting to four weeks for everyone. They do not establish that the two regimens are equivalent across every subgroup; identifying which children need longer treatment remains an open research question [28]. These trials were small and largely from one Australian research network, and the Cochrane review judged the certainty of evidence as moderate for short-term cure and low for adverse events, with no long-term outcome data assessable [27]; the evidence therefore supports a benefit of antibiotics for cough resolution with moderate certainty for short-term outcomes, and evidence that treatment prevents bronchiectasis is lacking.

Therapeutic approach

First-Line Antibiotic

Oral amoxicillin-clavulanate is the usual first-line empirical treatment. A commonly recommended regimen is 22.5-25 mg/kg per dose of the amoxicillin component, administered twice daily, with a maximum of 875 mg of amoxicillin per dose, initially for 14 days. The Royal Children's Hospital guideline uses 22.5 mg/kg per dose, and the Perth Children's Hospital guideline uses 25 mg/kg per dose [7,8]; the DACS trial used 25-35 mg/kg twice daily [28]. Formulations differ in amoxicillin-to-clavulanate ratio (for example, 7:1 vs 4:1); because clavulanate is the main driver of diarrhea, the ratio affects tolerability as well as dosing, and doses should be calculated from the amoxicillin component. DACS used a 7:1 suspension [28]. These are reference regimens for clinicians, not interchangeable liquid volumes. Before prescribing, verify the formulation, age, weight, renal function, and allergy history.

Review After Two Weeks

If the wet cough has resolved, complete the prescribed course and provide the family with a clear plan for return. If not, check adherence, dosing, adverse effects, and whether the diagnosis still fits. Extension of the same antibiotic to a total of four weeks can be considered when the cough has improved but not resolved after two weeks of confirmed adherence to an appropriate dose, the child remains well, and no warning signs are present. Children with no improvement, deterioration, or new warning signs should not be extended automatically but investigated. If the cough persists after four weeks of appropriate treatment, proceed to further investigation.

Penicillin Allergy and Alternative Treatment

Take a careful allergy history, distinguishing intolerance from low-risk delayed rash, immediate hypersensitivity, and severe delayed reactions. Perth guidance lists oral cefuroxime for selected low-risk penicillin allergies and cotrimoxazole for high-risk allergies [8]; the choice depends on reaction, age, local susceptibility, and microbiology. Cotrimoxazole carries its own risk of severe cutaneous adverse reactions and should be used with caution, particularly in children with a history of severe drug reactions; this risk should be weighed against the original penicillin reaction and discussed with an allergy or infectious-disease specialist. Severe drug reactions require specialist advice. A macrolide should not automatically replace amoxicillin-clavulanate for convenience. When treatment fails repeatedly, it is better to clarify the diagnosis and obtain useful microbiology than to cycle through empirical antibiotic changes.

Antimicrobial Stewardship

Monitor gastrointestinal intolerance, rash, adherence, and cumulative antibiotic exposure. DACS found no significant difference in adverse events between treatment groups, but its sample size was too small to exclude uncommon harms or resolve questions about long-term resistance safety [28]. Stewardship here does not mean avoiding antibiotics altogether; it means treating a defined chronic wet-cough syndrome adequately while avoiding antibiotics for uncomplicated acute viral cough and indefinite treatment without diagnostic review.

Adjunctive Treatment

Bronchodilators and corticosteroids do not address the underlying bacterial process; therefore, they are only used when there is a separate indication, such as coexisting asthma. If wheeze improves but the wet cough persists, the treatment has failed. Airway-clearance techniques can be individualized for children with retained secretions, airway malacia, or established suppurative lung disease. However, intensive airway clearance, hypertonic saline, inhaled antibiotics, and long-term macrolides should not be extrapolated wholesale from bronchiectasis care to a straightforward, antibiotic-responsive PBB episode; bronchiectasis has its own treatment framework [22,29]. A 2025 Cochrane review of mucolytics for children with CSLD found only one small trial at high risk of bias, in non-cystic fibrosis bronchiectasis, and none in PBB [30].

Table 4 summarizes the checkpoints at which assessment and treatment should be reviewed.

**Table 4:** Assessment and treatment checkpoints PBB: protracted bacterial bronchitis The checkpoints were compiled by the authors from published pediatric guidance [5-8,19].

Checkpoint	Recommended action
Wet cough >4 weeks	Confirm continuity and wet character; examine the child; review growth and previous treatment.
Warning signs or systemic illness	Investigate or refer immediately according to severity; do not delay for a routine PBB trial.
Typical presentation without warning signs	Obtain baseline chronic-cough investigations as appropriate and begin a 14-day antibiotic course.
Cough resolved at review	Complete treatment, document resolution, and explain when to return.
Still wet at two weeks	Reassess diagnosis and treatment delivery; selectively extend to four weeks.
Still wet after four weeks, or frequent recurrence	Refer for targeted respiratory investigation rather than continuing empirical treatment indefinitely.

This pathway pulls together current pediatric cough guidance [5-8,19]; clinical deterioration always overrides the scheduled checkpoints.

Recurrent PBB and targeted investigation

More than three episodes in 12 months is the usual referral threshold [7,8], but referral does not need to wait for a fourth episode when attacks are severe, recovery is incomplete, or other concerns exist. Investigation should target the suspected problem rather than run an indiscriminate panel. This might mean a swallowing assessment for feeding-associated cough, sweat testing when the picture fits cystic fibrosis, immunological testing for unusual infection susceptibility, or specialized assessment for primary ciliary dyskinesia when the history is compatible. Bronchoscopy and CT are chosen based on evidence of obstruction, persistent infection, or structural lung disease. Once bronchiectasis is diagnosed, management should move beyond repeated PBB prescriptions toward structured respiratory care, microbiology-guided treatment, individualized airway clearance, and longitudinal monitoring [22,29].

Prognosis and relationship with bronchiectasis

The path from an isolated PBB episode to persistent suppurative lung disease and, for some children, bronchiectasis is increasingly framed within a broader pediatric bronchiectasis diagnosis and treatment framework, including addressing individually identifiable "treatable traits" [22,29]. In a prospective cohort of 161 children with PBB, 13 (8.1%) were diagnosed with bronchiectasis over a median follow-up of 25 months (IQR, 24-28). Recurrent PBB and lower-airway *H. influenzae *carriage carried greater risk; the hazard ratio for *H. influenzae* was 7.55 (95% confidence interval 1.66-34.28); a wide interval [9]. These are risk markers, not evidence of inevitable progression. A separate five-year study found bronchiectasis in 9.6% of children, again linking risk to early recurrence and *H. influenzae* on lavage [20]. Both cohorts were clinically selected, and imaging was performed only when indicated, so these figures should not be treated as universal estimates for every child diagnosed in primary care.

A 2025 German follow-up study of 62 children (of 200 eligible) assessed a median of 7.7 years after PBB found chronic or recurrent wet cough in 17.7% and at least one abnormal spirometry parameter in 24.2% [31]. Its small, single-center sample, incomplete participation, and lack of a control group limit conclusions about causation, but it reinforces the case for reassessing children with ongoing symptoms rather than assuming all early PBB is inconsequential. The evidence supports vigilance, not the claim that every episode progresses to bronchiectasis. No randomized trial has shown that antibiotic treatment prevents future bronchiectasis. The evidence most clearly supports that treatment improves cough and clears the current infection.

Research priorities

Key open questions include which children actually benefit from extended antibiotic courses, how to improve noninvasive microbiological sampling, and what reliably predicts recurrence and structural disease. Biofilm-directed treatments remain a research possibility rather than established care [13]. Future trials should track sustained recovery, antibiotic exposure, adverse effects, and long-term respiratory outcomes, not just short-term cough improvement [13,23,28]. A published core outcome set for PBB trials recommends daily-recorded cough resolution, relapse, and cumulative antibiotic use over at least 12 months, quality of life, antibiotic resistance, bronchiectasis on clinically indicated CT, and microbiological clearance when samples are available [32].

Limitations

This narrative review has several limitations. Only PubMed was systematically searched; Embase and the Cochrane Library were not, so relevant studies may have been missed. Study selection was performed by one author with review by a second author rather than by independent duplicate screening, and no formal risk-of-bias or Grading of Recommendations Assessment, Development and Evaluation (GRADE) assessment was applied. The evidence for antibiotic treatment derives from a small number of trials, largely from one research network, and evidence that treatment itself prevents bronchiectasis remains limited. Most data originate from high-income settings, particularly Australia, so generalizability to low-resource settings, where tuberculosis, HIV, and pertussis alter the differential diagnosis, is limited. Risk factors such as daycare attendance, tobacco smoke exposure, and socioeconomic and Indigenous disparities may also influence presentation and outcomes.

## Conclusions

PBB should come to mind whenever a child has a daily wet cough lasting more than four weeks, especially without a more likely alternative diagnosis. Managing it well means an appropriate antibiotic course, reassessment, and documented resolution. Persistent symptoms after four weeks of adequate treatment, recurrent episodes, or warning signs all require investigation of an underlying disorder. The mistake to avoid is not only undertreatment but also repeatedly treating an unresolved wet cough without reconsidering the diagnosis.
